# Slow-flow, high-impact: radiologic features in venous malformations of the female genital tract

**DOI:** 10.1186/s13244-026-02343-z

**Published:** 2026-06-30

**Authors:** Maëlle Guihard, Antoine Fraissenon, Sophie Collardeau-Frachon, Pierre-Paul Bringuier, Pierre-Adrien Bolze, Laurent Guibaud, Pascal Rousset

**Affiliations:** 1 Department of Radiology, Hospices Civils de Lyon, Croix-Rousse Hospital, Université Claude Bernard Lyon 1, Lyon, France; 2 Department of Fetal and Pediatric Imaging, Expert Reference Center for Rare Diseases « Anomalies Vasculaires Superficielles », Hospices Civils de Lyon, Femme-Mère-Enfant Hospital, Université Claude Bernard Lyon 1, Lyon-Bron, France; 3 Departement of Pediatric Imaging, Nord University Hospital, Université Jean Monnet, Saint Etienne, France; 4 INSERM U1151 « Mécanismes et stratégies thérapeutiques dans les syndromes de surcroissance et les anomalies vasculaires », Institut Necker Enfants Malades, Paris, France; 5 Department of Pathology, Hospices Civils de Lyon, Groupement Hospitalier Est, Université Claude Bernard Lyon 1, Lyon-Bron, France; 6 Department of Gynecological and Oncological Surgery, Obstetrics, Hospices Civils de Lyon, Lyon Sud Hospital, Université Claude Bernard Lyon 1, Faculté de Médecine et de Maïeutique Lyon Sud - Charles Mérieux, CICLY, Lyon, France; 7 Department of Medical and Interventional Imaging, Hospices Civils de Lyon, Lyon Sud Hospital, Université Claude Bernard Lyon 1, Faculté de Médecine et de Maïeutique Lyon Sud - Charles Mérieux, CICLY, Lyon, France

**Keywords:** Vascular malformations, Hemangioma, cavernous, Genitalia, female, Magnetic resonance imaging, Ultrasonography

## Abstract

**Abstract:**

Gynaecological venous malformations are rare, congenital slow-flow vascular anomalies arising from early developmental errors. They may occur as isolated lesions or within syndromic contexts (associated with somatic gene variants such as *PIK3CA* and *TEK*). They remain poorly documented and frequently misclassified due to persistent outdated terminology and diagnostic challenges—requiring integration of clinical, radiological, and histopathological data. Imaging plays a pivotal role in the diagnosis of gynaecological venous malformations: ultrasound serves as the first-line imaging modality, while Magnetic Resonance Imaging offers superior anatomical detail, confirms the diagnosis, and guides treatment planning and follow-up. However, the lack of standardised protocols and consensual imaging features complicates differentiation from other gynaecological vascular conditions with overlapping radiological appearances. Given diagnostic challenges and concerns regarding fertility, a multidisciplinary approach in specialised vascular anomaly centres combining clinical, radiological, histological, and genetic data is essential. This review aims to provide a complete sonographic and MRI framework aligned with current nomenclature, with key clinical and histopathological insights to support radiologists in their diagnostic process.

**Critical relevance statement:**

Gynaecological venous malformations are frequently overlooked or misdiagnosed due to shared imaging features and a lack of standardised protocols, making accurate imaging description and multidisciplinary assessment essential for a positive diagnosis and appropriate management in dedicated centres.

**Key Points:**

- Gynaecological venous malformations are rare and often misdiagnosed congenital anomalies.
- Diagnostic features include an organ enlargement with a sponge-like appearance and slow-flow.

**Graphical Abstract:**

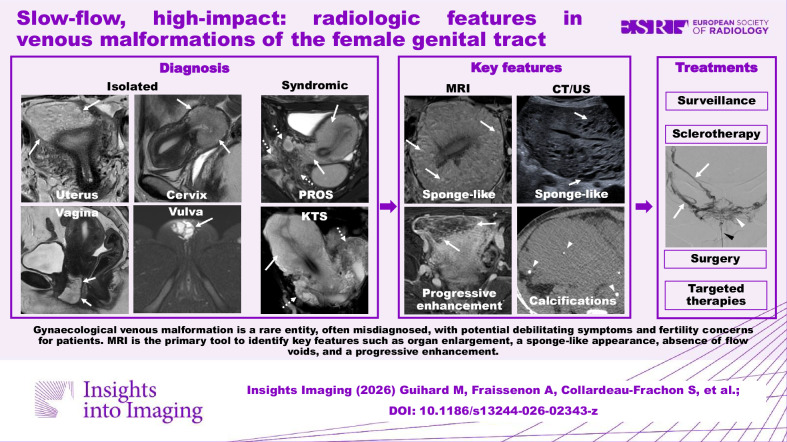

## Introduction

Vascular malformations are divided into fast-flow (with an arterial component) and slow-flow types (venous, capillary, and lymphatic) by the International Society for the Study of Vascular Anomalies (ISSVA) in its 2025 updates [1]. They can affect a wide range of organs, including the gynaecological system. Slow-flow types are purely congenital, while fast-flow types—such as arteriovenous fistulas—may also be acquired. Fast-flow malformations are relatively well-known entities, arising from broader genetic syndromes or following iatrogenic or traumatic events. Conversely, slow-flow gynaecological malformations, particularly venous ones, are exceedingly rare, poorly documented in the literature, and often misdiagnosed.

Indeed, the exact prevalence of gynaecological venous malformations (GVMs), whether located in the uterine body, cervix, vagina or vulva, and more rarely, the adnexa, remains unclear due to scarce data [2, 3]. Likewise, their imaging characteristics are not well established, in contrast to better-known cutaneous venous malformations. Nevertheless, imaging plays a crucial role in diagnosis, treatment planning and optimisation of patient care, notably in young women and regarding fertility outcome.

Despite recent updates and refinements in classification systems, the terminology used in the literature to describe GVMs remains particularly heterogeneous and confusing. Several radiological case reports continue to use the outdated term “cavernous haemangioma”, conflating malformations and tumours, and blurring distinctions between entities with different clinical and pathological behaviours [4, 5]. Moreover, while older WHO classifications included these terms, more recent versions have phased them out, reflecting an ongoing evolution in nomenclature and contributing to variability across classifications [1, 6].

Histological differentiation between vascular tumours and malformations—or between venous and arteriovenous lesions—can be challenging, especially when only limited biopsy material is available, as partial sampling increases the risk of misinterpretation. This diagnostic uncertainty likely contributes to the lack of consensus regarding the optimal therapeutic approach for patients. Nevertheless, histological examination is often essential to confirm the vascular nature of the lesion and to rule out differential diagnoses. Furthermore, tissue sampling is required for molecular analysis, which may guide the use of targeted anti-angiogenic therapy in selected cases. These considerations highlight the importance of a multidisciplinary approach integrating clinical, radiological, and pathological data to ensure a comprehensive and accurate interpretation [4]. Moreover, GVMs may occur in isolation or within a broader syndromic framework, frequently associated with genetic anomalies. These associations open possibilities for new promising targeted therapies, but systematic screening for these conditions is not yet standard practice, limiting personalised care options.

As a rare but impactful pathology, this review aims to provide a comprehensive ultrasonography (US) and MRI framework based on the current nomenclature, emphasising “positive” and “negative” imaging signs, key clinical and histopathological insights, features suggestive of syndromic involvement, and differential imaging features of other vascular gynaecological conditions to assist radiologists in their diagnostic process.

## Epidemiology and clinical characteristics

The exact prevalence of GVMs remains uncertain due to limited available data, and, to our knowledge, no robust epidemiological estimates are currently available [2, 3]. The literature primarily consists of case reports or small case series, many of which still rely on outdated or inconsistent terminology.

GVMs can be diagnosed at any age, from the prepubertal to postmenopausal period, without significant predominance [2, 7, 8]. The most affected site is the vulva (especially the labia majora) in superficial localisations [9]. Deeper lesions are more frequently seen within the uterine myometrium, often involving all layers of the uterine wall diffusely [10]. GVMs in deep pelvic structures such as the cervix or uterus are frequently underdiagnosed and/or with a delayed diagnosis. Conversely, superficial vulvar or vaginal lesions may be detected earlier during dermatological or gynaecological examinations, often presenting as bluish discolouration and gravity-dependent swelling [9, 11].

Clinical presentation ranges from asymptomatic forms to chronic abnormal gynaecological bleeding (e.g., menometrorrhagia) regarding uterine or cervical GVMs, including in premenopausal and postmenopausal individuals [2, 7, 8]. Other signs include chronic pelvic heaviness, pain, or acute inflammatory episodes related to intralesional thrombosis, as seen in other venous malformation localisations [11]. These features are important diagnostic clues that should not be overlooked.

Additional manifestations may include anaemia, dysmenorrhoea, or potential fertility impact, although some cases have resulted in successful spontaneous pregnancies [2]. Extensive venous malformations are frequently associated with localised intravascular coagulation, expressed as increased systemic D-dimers and reduced fibrinogen levels [9, 11, 12]. This coagulopathy can also occur in GVMs: during pregnancy, disseminated intravascular coagulation or postpartum haemorrhage (due to impaired dysplastic vessel contraction) have been reported [2, 13].

## Histopathology and pathogenesis

Gynaecological venous malformations are characterised by dysmorphic vessels and result from errors during early vascular development [11, 14]. These anomalies are driven by genetic mutations—most often somatic, though sometimes germline—that impair intracellular signalling pathways [11, 15].

They consist of excessive and/or malformed ectatic venous channels that vary in calibre, number, shape, and degree of muscularization, frequently showing thrombotic changes, with varying degrees of endothelial proliferation—though less pronounced than in vascular tumours [4]. Phleboliths, which are calcified thrombi, are considered pathognomonic and are present in nearly 50% of cases [11]. These ectatic changes likely explain the historical use of the term “cavernous haemangioma” for this entity, often causing confusion as “haemangioma” commonly refers to vascular tumours. This term has now been excluded from the ISSVA 2025 update [1] and the WHO 2023 classification of Paediatric Tumours [6].

Venous malformations can be further categorised into several types based on their connection to the normal systemic venous network, which may influence therapeutic decision-making depending on their type [16, 17].

Many radiological case reports of GVMs still use the term “haemangioma”, which falsely implies a proliferative process, suggesting that most cases are of acquired origin, often attributed to physical or hormonal changes [2, 7]. Although such factors may reactivate or unmask an underlying venous malformation [3, 14, 18], they are not the original cause, and using such a term falsely suggests a single underlying entity. Caution is therefore warranted when interpreting the literature, highlighting the importance of a critical reading to identify specific criteria that may help differentiate vascular tumours from malformations.

Nonetheless, histological diagnosis of gynaecological venous malformations can be challenging, particularly in small biopsy samples and in the absence of specialised expertise. The overlapping features between different lesions, combined with ambiguous or outdated terminology, may lead to misinterpretation. Therefore, accurate diagnosis requires a multidisciplinary approach, combining histopathological findings with clinical, radiological, and molecular data, preferably in a dedicated expert centre for vascular anomalies.

## Genetic and syndromic involvement

Venous malformations can occur as isolated conditions or as part of broader syndromes, often characterised by segmental overgrowth and musculoskeletal, cutaneous, or neurologic abnormalities related to genetic variants arising during embryogenesis [19]. The earlier the mutation occurs, the more extensive and complex the lesion can be, due to the pluripotency of the affected cells. In contrast, postnatal mutations result in the growth of cells from the mutated tissue into a localised tumour [4].

Several syndromes are associated with GVMs, some of which fall under the PIK3CA-Related Overgrowth Spectrum (PROS). These conditions are caused by post-zygotic somatic variants in the *PIK3CA* gene, creating a mosaicism and subsequent hyperactivation of the PI3K-AKT-mTOR pathway, which promotes endothelial proliferation and growth—a hallmark of most slow-flow vascular malformations [4, 19–21]. Many disorders including venous malformations, such as CLOVES [22] or Klippel-Trénaunay syndrome [23], have been linked to *PIK3CA* mosaic mutations [24].

The identification of these molecular pathways has paved the way for targeted therapies such as alpelisib (targeting PI3K) and miransertib (inhibiting AKT, a downstream product), both showing promising results in symptom management [19, 21].

## Imaging techniques

### Ultrasonography

Ultrasound is the first-line imaging technique for detecting and characterising vascular anomalies [12, 14, 17, 25]. Transabdominal ultrasonography should be performed with a full bladder, and, when feasible, transvaginal assessment is recommended for GVMs. In the case of GVMs, particularly for large or extensive deep disease, the limited field of view and operator experience may limit exploration, providing only a partial evaluation.

### Magnetic resonance imaging

Thanks to its high specificity in tissue characterisation and large field of view, MRI is a key imaging technique. It enables the precise definition of vascular lesions and their anatomical relationship with adjacent structures [17]. MRI is also the preferred imaging technique for pre-procedural diagnosis, interventional planning, and post-procedural follow-up of venous malformations [12]. There is no standardised MR protocol for GVMs in the literature. However, as for any venous malformation, it should include at least two orthogonal T2-weighted sequences and T2 fat-suppressed (FS) sequences—a key sequence to highlight veins—and axial T1-weighted sequences [12, 17]. Pre- and post-gadolinium injection T1W FS sequences can help differentiate vascular malformations from vascular tumours by assessing contrast uptake, with dynamic sequences being a useful tool for precise analysis of the involved vessels to differentiate fast-flow from slow-flow lesions [17]. Additionally, DWI sequences may aid in identifying an underlying tumour. The MR protocol used in our centre is proposed in Table 1.

**Table 1:** Dedicated MRI protocol for suspected gynaecological venous malformations (GVM)

Sequence	Plane	Usefulness for GVM diagnosis
T2-weighted	At least axial and sagittal	Anatomical description and localisation of the lesion Possible flow-voids and absence of solid components
STIR or T2-weighted fat-suppressed *Key sequence in the diagnosis of vascular malformations*	At least axial and coronal	Slow-flow venous lesion Lesion extent and relation with adjacent structures
T1 DIXON or T1-weighted fat-saturated before gadolinium enhancement	Axial	Intralesional changes (blood, phleboliths)
DWI *Optional if a solid component is suspected or uncertain*	Axial	No solid component No restriction on ADC map
Dynamic gadolinium-enhanced T1 DIXON *Useful at initial diagnosis or in cases of equivocal lesions*	Axial or sagittal	Progressive enhancement pattern and absence of solid component
Late gadolinium-enhanced T1 DIXON or T1-weighted fat-saturated	Axial	Complete and homogenous or incomplete and heterogeneous, patchy delayed enhancement

### Computed tomography

CT plays a limited role due to its low contrast resolution for such entities. Moreover, in accordance with the ALARA principle (As Low As Reasonably Achievable), particularly in young women of reproductive age, the use of CT should be avoided and only considered when MRI is not tolerated or is contraindicated.

## Imaging features of gynaecological venous malformations

### Sporadic and isolated involvement

GVMs may manifest as isolated anomalies arising from defects during early vascular development, linked to sporadic genetic mutations. Various gynaecological locations have been reported, including the uterine body and cervix, vagina and vulva [2, 3, 9]. Adnexal location remains possible, although no cases have been published to date, to the best of our knowledge. Although imaging descriptions are scarce, the main findings reported across different modalities are fairly consistent, offering both positive and negative signs that guide diagnostic assessment (Table 2) [17, 26].

**Table 2:** Overview of imaging characteristics of gynaecological venous malformations across modalities

Radiological findings	Common features	Ultrasonography	MRI	CT
Organ enlargement	Global and diffuse/focal and well-delimited thickening of the organ’s tissues	Heterogenous Hypoechoic or hyperechoic Compressible if superficial (vulva, vaginal walls)	T2 and T2 FS hyperintense T1 iso or hypointense Mild DWI signal with no restriction	Non-specific, poorly defined aspect Iso/hypodense on nonenhanced CT
Sponge-like sign	Tubular, serpiginous or lobulated appearance corresponding to a network of veins of varying sizes	Hypoechoic or anechoic structures within the lesion Hyperechoic in the case of small vascular channels	T2 and T2 FS hyperintense with a subtle lower, heterogeneous signal defining the vascular walls within the lesion	Not applicable
Vascular flow	Slow-flow pattern	Monophasic venous flow No aliasing No flow in 16% of cases Accentuated with Valsalva techniques if superficial	No flow-voids	Not applicable
Contrast enhancement	Progressive, variable enhancing pattern (homogeneous to heterogeneous) No early arterial enhancing pattern nor nidus	Not applicable	Slow and progressive on dynamic sequences Homogeneous or heterogeneous on delayed sequences	Gradual enhancement often heterogeneous and delayed
Phleboliths/thrombi	Variable	Hyperechoic structure with acoustic shadowing cone (phleboliths) Echogenic, non-compressible image within the lesion with no flow on Doppler imaging (thrombi)	T1 and T2 focal dark spot, blooming artefact on gradient echo sequence, no enhancement (phleboliths) T1 and T2 hypersignal, T2 hyposignal if chronic, no enhancement (thrombi)	Small, round or oval calcified focus within the lesion (phleboliths) Spontaneously dense, non-enhancing image within the lesion (thrombi)

#### Ultrasonography

**Fig. 1:**
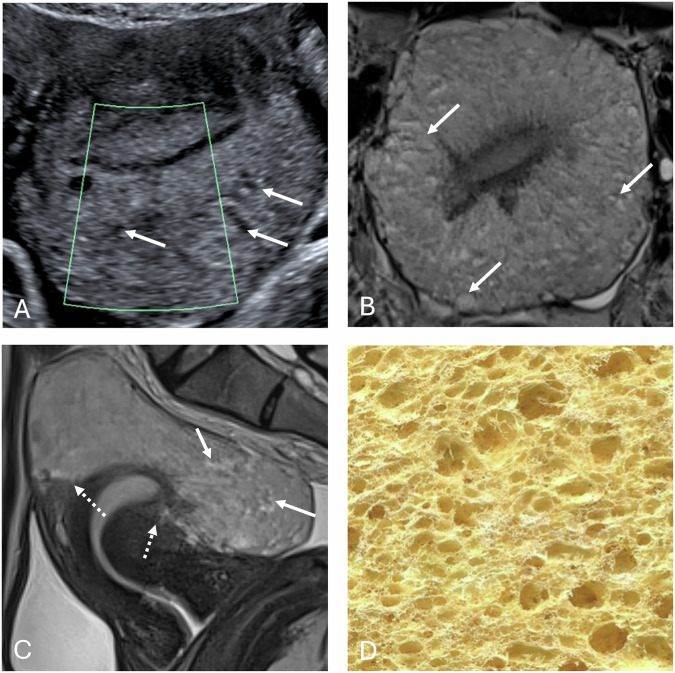
Large uterine gynaecological venous malformation (GVM) in a 34-year-old patient, G2P2, with new-onset abdominal pain. Surgical biopsy confirmed the diagnosis of GVM. **A** Suprapubic ultrasonography image demonstrates a large myometrial thickening of the uterine fundus with multiple scattered hypoechoic or anechoic tubular structures (arrows) and some hyperechoic foci. Note the absence of Doppler signal. **B** Axial and **C** sagittal T2-W MR images show a lobulated T2-hyperintense thickening of the uterine fundus, sharply demarcated from the normal myometrium (dotted arrows), containing serpiginous or tubular structures of variable size and signal intensity with a sponge-like pattern (arrows). Note the absence of flow-voids. **D** Photograph of a sponge texture illustrating a pattern resembling that of the GVM

**Fig. 2:**
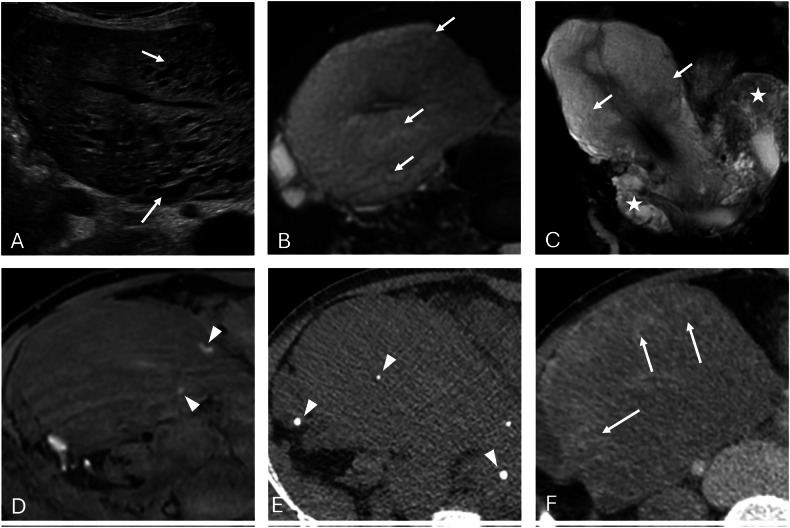
Multiple venous malformations of the gynaecological tract, bowel, soft tissues, and lower limbs in a 46-year-old patient with Klippel-Trénaunay syndrome and a PIK3CA mutation. **A** Suprapubic ultrasonography image shows a diffuse myometrial enlargement with multiple anechoic myometrial serpiginous structures (arrows). **B** Axial and **C** coronal T2-W fat-suppressed MR images show marked uteromegaly with a diffuse T2-hyperintense thickening of the myometrium and a sponge-like pattern (arrows). Note the similar enlargement of the adjacent affected bowel loops (stars). **D** Axial T1-W fat-suppressed MR image shows some scattered hyperintense areas (arrowheads) corresponding to focal thrombi within the venous malformation. **E** Axial non-contrast CT image shows scattered calcifications within the myometrium and bowel loops, corresponding to tiny phleboliths (arrowheads). **F** Axial post-contrast CT image shows minimal and delayed peripheral enhancement of the uterine venous malformation (arrows)

**Fig. 3:**
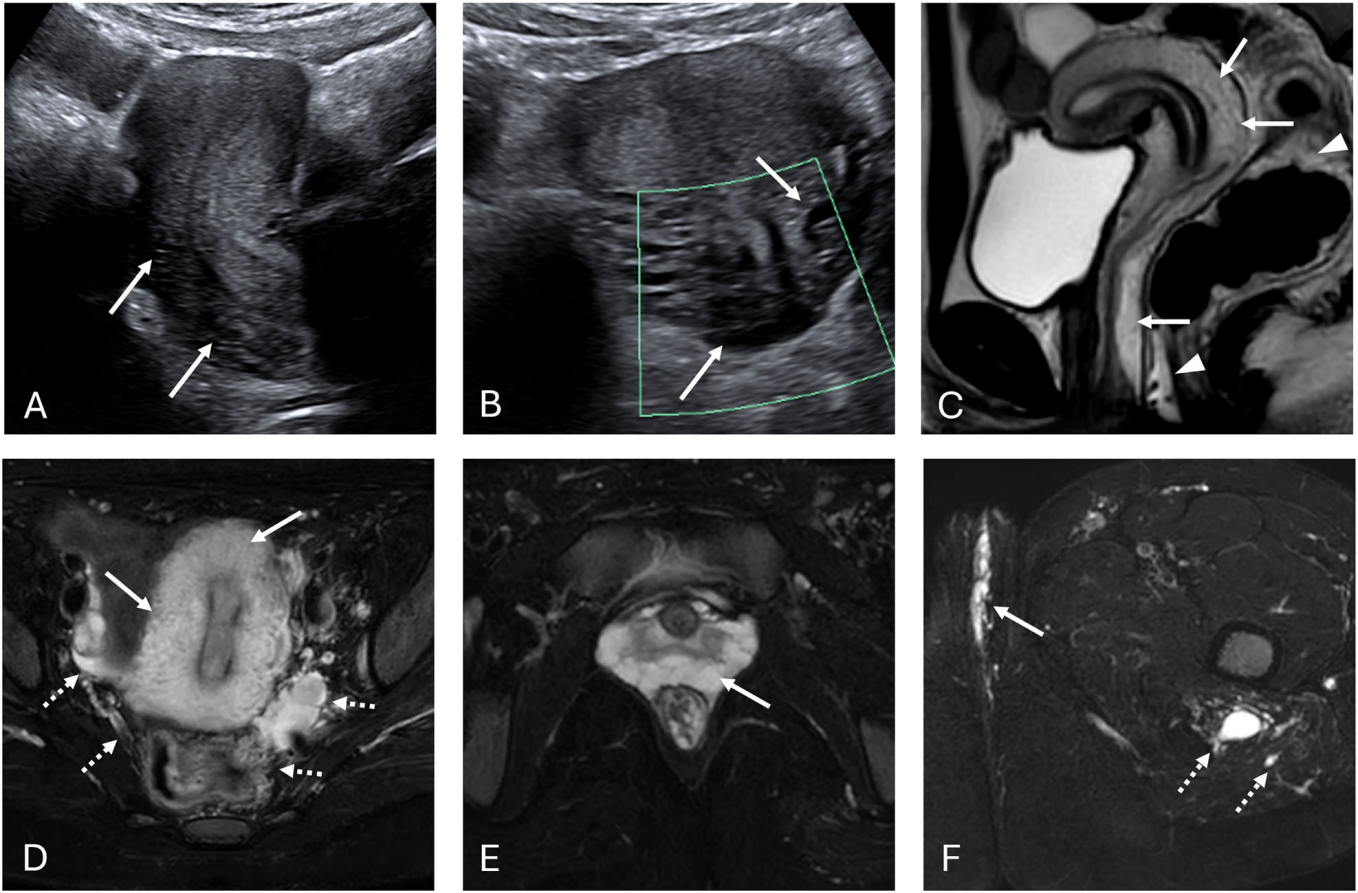
Extensive venous malformations involving the female genital tract in a 13-year-old patient with PROS syndrome and a PIK3CA mutation, treated by alpelisib. **A**, **B** Suprapubic ultrasonography images show hypoechoic lacunae within the myometrium and cervix (arrows), with no Doppler signal. **C** Sagittal T2-W MR images show diffuse T2-hyperintense thickening of the uterine corpus, cervix and posterior vaginal wall (arrows), with a sponge-like pattern, better appreciated than on suprapubic ultrasonography. Note the similar involvement of the rectum and anal canal, indicative of a multi-organ disease (arrowheads). **D** At the uterine level, **E** at the vaginal level and **F** at the perineal and left thigh level, axial T2-W fat-suppressed MR images show diffuse T2-hyperintense thickening of the uterine body, vaginal walls and vulva (arrows). Note the similar involvement of the adjacent pelvic tissues and lower limb, with serpiginous dilated venous structures (dotted arrows) and no visible flow-voids

**Fig. 4:**
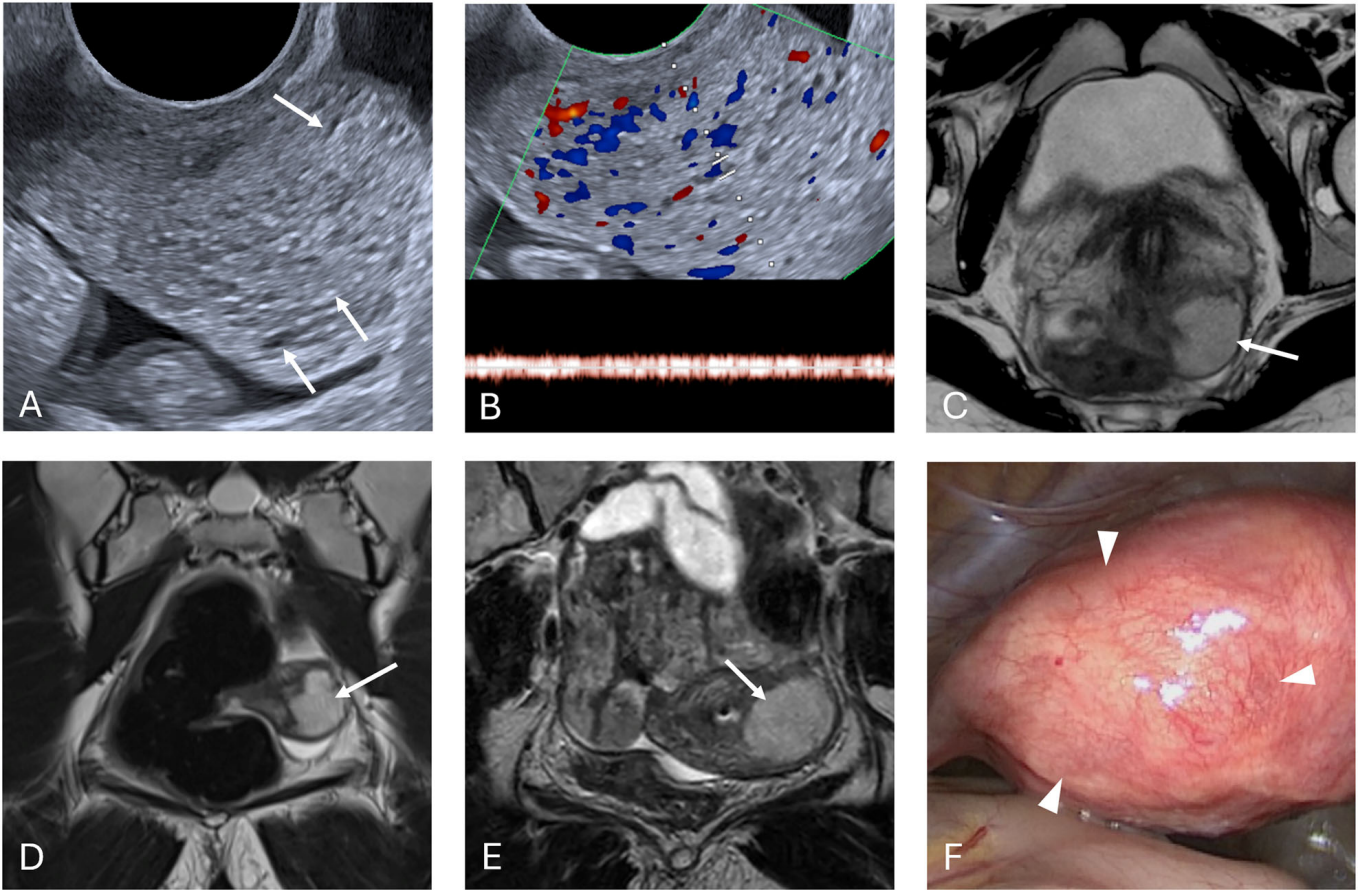
Fundal gynaecological venous malformation (GVM) in a 22-year-old patient, G0P0, presenting with secondary amenorrhoea after discontinuation of oral contraceptives and normal hysteroscopic findings. **A** Transvaginal ultrasonography image shows a hyperechoic, heterogeneous fundal lesion with hypoechoic tubular structures and hyperechoic foci (arrows). **B** Transvaginal ultrasonography image performed without Valsalva manoeuvres and using colour Doppler imaging shows slow, monophasic venous flow with a measured velocity of 5 cm/s. **C** Axial and **D** coronal T2-W MR images show a well-defined, focal left-sided T2-hyperintense thickening of the uterine fundus myometrium, with a homogeneous appearance (arrows). **E** Coronal T2-W MR image obtained 1 year later shows a slight increase in lesion size, with a more heterogeneous internal signal and a more marked sponge-like pattern (arrow). **F** Intraoperative laparoscopic photograph shows a focal subserosal fundal bulge, consistent with the MR findings (arrowheads). Histopathological analysis of the surgical biopsy confirmed the diagnosis of GVM. Percutaneous transgluteal sclerotherapy was attempted but was unsuccessful due to the absence of lesion opacification

GVMs may enlarge and alter the normal zonal anatomy of the involved structure. They appear as diffuse or localised thickening of the myometrium, cervix, or vaginal walls, often associated with multiple hypoechoic or anechoic compressible tubular structures or less organised hypoechoic lacunae, corresponding to a network of veins of different sizes, with a sponge-like appearance (Figs. 1–3, Supplementary Fig. 1) [13, 27]. These may sometimes be punctuated by phleboliths, seen as echogenic foci with posterior acoustic shadowing [12, 14, 25, 28]. Colour Doppler allows the detection of a key diagnostic feature by demonstrating either low monophasic venous flow (with velocities around 5 cm/s) within the multi-tubular structures, or more rarely an absence of flow (Figs. 1, 3, 4) [12, 14, 25, 28]. During the Valsalva manoeuvre, colour Doppler can also show an enhanced venous flow [3, 12].

A heterogeneous, hyperechoic lesion appearance can occasionally be observed, likely due to the presence of smaller, less ectatic vessels, along with increased ultrasound repetition artefacts at greater exploration depths (Figs. 1, 4), as previously described in cutaneous venous malformations, where lesions with small vascular channels are more echogenic and less compressible than those with large vascular channels [29].

#### Magnetic resonance imaging

**Fig. 5:**
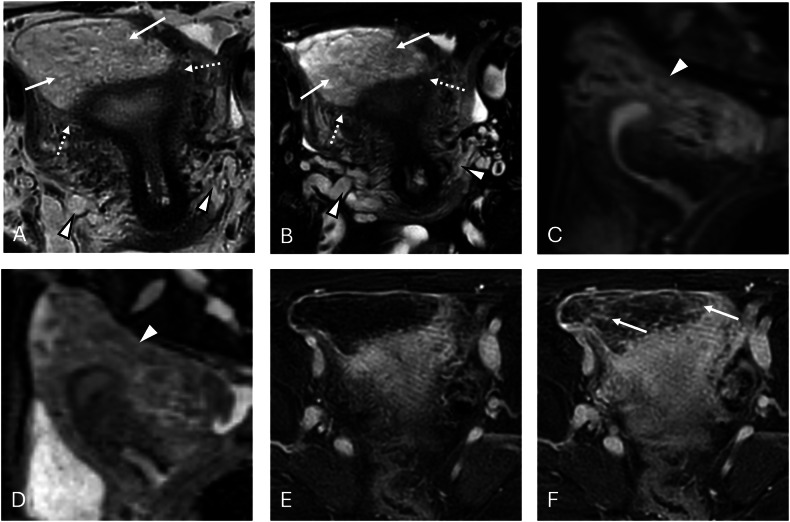
Gynaecological venous malformation (GVM) confirmed by uterine biopsy in a 34-year-old patient and after a successful spontaneous pregnancy. **A** Axial T2-W and **B** T2-W fat-suppressed MR images show a well-defined, T2-hyperintense fundal lesion with a sponge-like appearance (arrows), sharply demarcated from the adjacent normal myometrium (dotted arrows). Parametrial venous varicosities are present bilaterally (arrowheads), a finding that may be associated with GVM but is non-specific; in this patient, these were not present on pre-pregnancy MRI, suggesting venous insufficiency. **C** Sagittal diffusion-weighted imaging (DWI) and **D** apparent diffusion coefficient (ADC) map MR images show no diffusion restriction (arrowheads). **E** Axial dynamic post-contrast T1-W fat-suppressed MR images at arterial and **F** at venous phase show slow and progressive peripheral enhancement of the lesion (arrows) relative to adjacent myometrium, without early enhancement, nidus, or solid tissue component, supporting slow venous flow

**Fig. 6:**
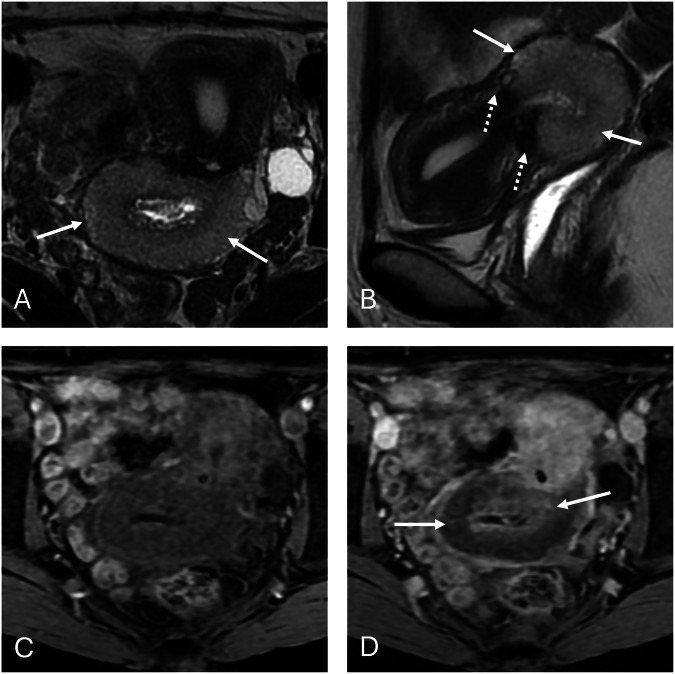
Cervical gynaecological venous malformation (GVM) in a 48-year-old patient, detected on clinical examination as cervical enlargement, raising suspicion of neoplasm. **A** Axial and **B** sagittal T2-W MR images show a well-defined, circumferential T2-hyperintense thickening of the cervix with a subtle sponge-like appearance (arrows), with no infiltrative pattern. Note the absence of flow-voids and the sharp delineation (dotted arrows) from the uterine isthmus and body, which appear normal. **C** Axial dynamic post-contrast T1-W fat-suppressed MR images at the arterial and **D** at the venous phase show a slow, progressive enhancement of the lesion (arrows), consistent with slow venous flow, without any arterial component

**Fig. 7:**
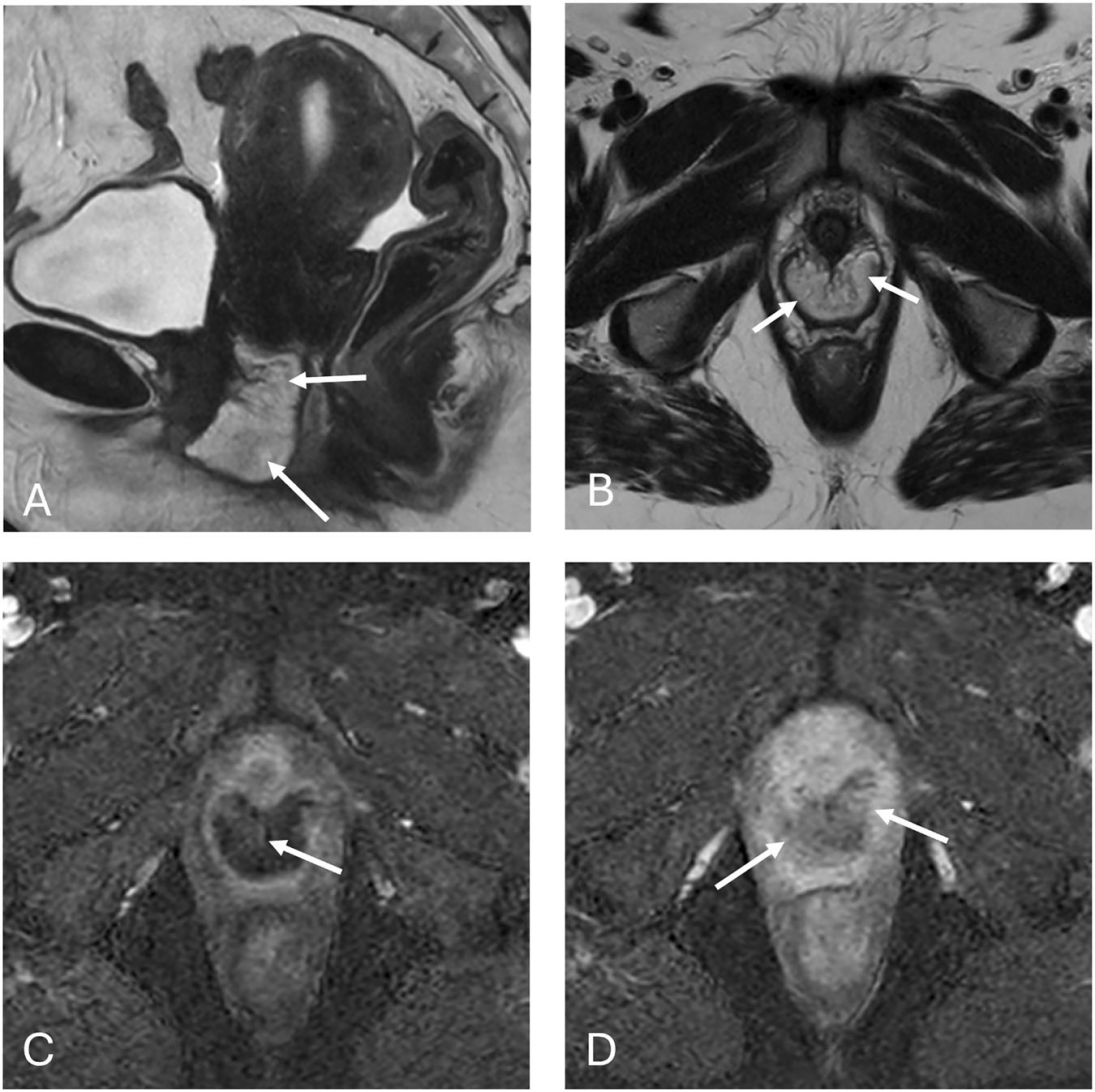
Vaginal gynaecological venous malformation (GVM) in a 48-year-old patient, G4P4, presenting with significant vaginal bleeding and a focal mass of the anterior vaginal wall identified several decades earlier during her first pregnancy. **A** Sagittal and **B** axial T2-W MR images show a well-defined thickening of the anterior vaginal wall, displaying T2-hyperintense signal (arrows). The rest of the genital tract appears normal. **C** Axial dynamic post-contrast T1-W fat-suppressed MR images at the arterial and **D** at the venous phase demonstrate slow, progressive enhancement of the lesion (arrows), without nidus or solid tissue component, suggestive of slow venous flow. Vaginal biopsy confirmed the diagnosis of GVM and identified a PIK3CA mutation

**Fig. 8:**
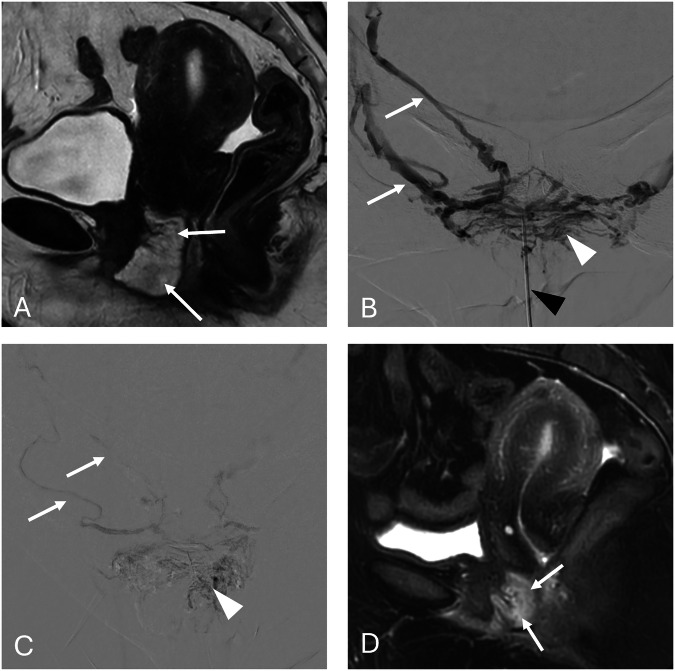
Vaginal gynaecological venous malformation (GVM) treated by transperineal sclerotherapy in a context of severe and persistent vaginal bleeding. **A** Sagittal T2-W MR image obtained before treatment shows a well-defined, subtly heterogeneous T2-hyperintense lesion of the anterior vaginal wall (arrows), with no visible flow-voids and no involvement of the rest of the genital tract. **B** Coronal image of transperineal venography before sclerosing agent injection shows dilated dysplastic venous channels of the vaginal GVM (white arrowhead) with several draining veins (arrows), not retrospectively visible on MRI. As a transperineal procedure, a needle was directly inserted within the dysplastic vessels of the lesion (black arrowhead). **C** Coronal image of transperineal venography after injection of 5 mL of sclerosing agent shows a satisfactory result with minimal to no residual vascular flow in the vaginal GVM (white arrowhead) nor in draining veins (arrows). Sclerosing agent consisted of an emulsion of 2 mL of 3% aetoxisclerol, 0.8 mL of lipiodol, and 2 mL of air. **D** Sagittal T2-W fat-suppressed MR image obtained at 4-month follow-up shows a significant reduction in lesion size (arrows). The patient reported complete resolution of symptoms

**Fig. 9:**
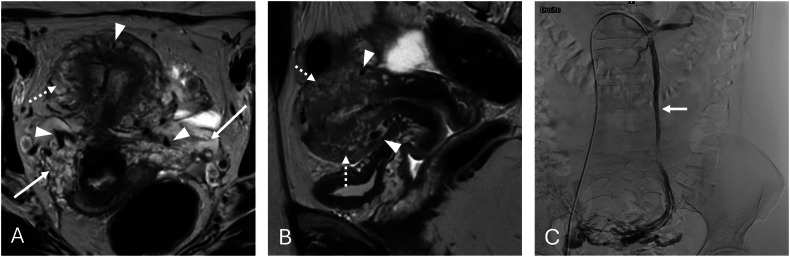
Pelvic congestion syndrome (PCS) in a 34-year-old patient presenting with dysmenorrhoea, dyspareunia, and pelvic pain, initially screened for endometriosis. **A** Axial and **B** sagittal T2-W MR images show diffuse T2-hyperintense venous varicosities of the outer myometrium (dotted arrows) with preservation of the zonal anatomy and normal appearance of the junctional zone, as well as predominantly left-sided venous varicosities in the parametria (arrows). The presence of some flow-voids (arrowheads) indicates faster venous flow. **C** Coronal image of venography with Valsalva manoeuvres demonstrates venous reflux into the left gonadal vein (arrow) associated with pelvic venous varicosities, confirming the clinical diagnosis of PCS

GVMs demonstrate imaging characteristics consistent with venous malformations in other anatomical regions. They can enlarge or deform the affected structure and alter the normal zonal anatomy, presenting as either a focal mass or diffuse infiltration of the myometrium (Figs. 1–6), cervix (Figs. 3, 7), vagina (Figs. 3, 8, 9) or vulva (Fig. 3, Supplementary Fig. 1). They typically appear as a well-defined, lobulated area, resembling a pseudomass enlarging anatomical segment and composed of serpiginous vascular structures (Figs. 1–9) [8, 9, 12–14, 17, 29].

**Fig. 10:**
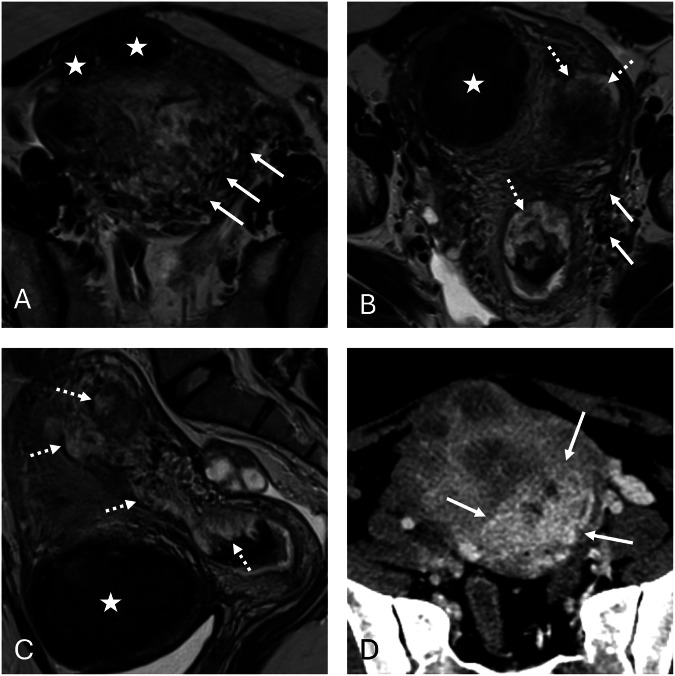
Gestational trophoblastic tumour (choriocarcinoma) in a 27-year-old, G2P2, presenting with postpartum metrorrhagia and elevated HCG. **A**, **B** Axial and **C** sagittal T2-W MR images show a poorly defined T2-hyperintense enlargement of the left corporeo-isthmic region of the uterus, with numerous flow-voids (arrows) and intracavitary tissue components invading the myometrium (dotted arrows). A vaginal metastasis was also visible (not shown). Incidental uterine fibroids are also present (stars). **D** Axial post-contrast CT image at the arterial phase shows early enhancement of a myometrial tissue infiltration with ill-defined margins (arrows)

On T2-weighted images, they appear hyperintense with persistent signal on fat-suppressed sequences—a key feature indicating their slow-flow vascular nature—(Figs. 2, 3, 5, 9), corresponding to a network of veins of different sizes, with a sponge-like appearance (Figs. 1–3, 5–7) [8, 12, 13, 17, 25, 29]. Smaller lesions or those with narrower vascular channels may exhibit a more homogeneous hyperintensity on T2-weighted sequences, likely due to spatial resolution limitations (Fig. 4) [29]. Unlike vascular tumours and arteriovenous malformations, they show no flow-voids because of their slow-flow type (Fig. 10) [12, 17]. Occasionally, tiny low-signal-intensity striations due to chronic thrombosed vessels or dark round phleboliths that are focal and do not show any enhancement on post-contrast sequences, unlike flow-voids that are serpiginous and enhance [12, 14, 17, 25].

On T1-weighted images, they appear hypointense or isointense, occasionally showing focal heterogeneous hyperintense areas (Fig. 2) in cases of focal bleeding with or without thrombosis, and occasionally with a linear hypersignal corresponding to thrombi [13, 17, 25, 28].

Post-contrast dynamic sequences typically show slow and gradual enhancement, often starting peripherally, without early arterial enhancement, and present either a homogeneous or heterogeneous appearance on later phases, possibly linked to the lesion’s size and time of acquisition (Figs. 5, 7, 8) [8, 9, 12, 13, 17, 25, 28, 29]. Post-contrast sequence is especially useful for initial lesion assessment or in cases of diagnostic uncertainty. DWI sequences are recommended in cases of diagnostic uncertainty for vascular tumours: mild hyperintensity may be seen on DWI due to the T2-shine-through effect, with no corresponding ADC reduction (Fig. 5).

In some cases, GVMs are accompanied by ectasia of neighbouring venous structures (Figs. 5, 6), which varies according to the venous drainage subtype; however, no enlarged feeding arteries or arteriovenous shunting are observed—compared to AVMs or vascular tumours [17]. While visualisation of a venous drainage seems very difficult on MRI compared to venography (Fig. 9), identifying a connection between the malformation and the systemic venous system is crucial for treatment planning, as such findings increase the risk of deep venous thrombosis [17] or toxicity following treatment [16, 29]. Conversely, the absence of evident venous drainage along with well-defined lesion margins on MR imaging has been shown to predict a favourable outcome after percutaneous sclerotherapy in superficial localisations [17].

#### Computed tomography

On CT imaging, uterine, cervical or vaginal involvement may appear as a focal mass or diffuse isodense tissue enlargement, sometimes with scattered calcifications corresponding to phleboliths and a lobulated, heterogeneously enhancing lesion aspect (Fig. 2), in line with the typical radiological features of venous malformations in other locations [9, 25, 26].

In syndromic forms, CT scan is not used for pelvic assessment but rather to search for additional information on potential bone involvement (hyperplasia or hypoplasia, scoliosis, etc.), or other soft tissue localisations, as well as identifying phleboliths [14].

### Syndromic involvement

When part of broader syndromes, GVMs appear in conjunction with segmental overgrowth and musculoskeletal, cutaneous or neurologic abnormalities (Table 3). Imaging findings typically demonstrate both deep involvement—potentially affecting additional organs—and superficial extension, most commonly involving the cutaneous tissues and unilateral involvement of the perineal region, external genitalia or lower extremities (Figs. 2, 3, Supplementary Fig. 2). The radiological presentation closely parallels that of isolated GVMs, characterised by diffuse or localised thickening of involved deep structures, exhibiting high signal intensity on T2-weighted sequences, isointensity on T1-weighted sequences, and progressive, delayed contrast enhancement. Superficial soft tissue involvement may appear as focal or diffuse serpiginous infiltration, with similar signal characteristics (Fig. 3, Supplementary Fig. 2).

**Table 3:** Vascular syndromes potentially involving gynaecological venous malformations

Syndrome	MRI features	Typical distribution	Associated findings	Genes	Somatic/germline
Klippel-Trénaunay syndrome (KTS)	· Slow-flow venous malformations · Persistent embryonic veins · Fat and muscle overgrowth · Sciatic nerve enlargement	Typically involves unilateral lower limb	· Capillary malformations · Limb hypertrophy · Lateral marginal vein	*PIK3CA*	Somatic (post-zygotic)
CLOVES syndrome	· Complex slow-flow malformations (venous and lymphatic) · Fat overgrowth · Epidural flow-voids · Skeletal asymmetry · Nerve enlargement	Trunk Extremities Spinal or paraspinal	· Lipomatous overgrowth · Scoliosis · Epidermal nevi · Spinal AVMs	*PIK3CA*	Somatic (early mosaic)
Cutaneo-mucosal venous malformation (VMCM)	· Multifocal well-circumscribed, compressible venous malformations	Skin Mucosa	· Pain · Swelling · Risk of thrombosis	TIE2 (*TEK*)	Germline (heterozygous), often with somatic second hit
Bockenheimer disease	· Diffuse, infiltrative venous malformation · Multiple-layer involvement from skin to bone	Typically involves the entire limb	· Pain · Swelling · Risk of thrombosis · Functional limitation	TIE2 (*TEK*)	Somatic (early mosaic)

In this context, GVMs are less likely to be overlooked when accompanied by more recognisable locoregional venous anomalies in other anatomical regions, which help support the diagnosis: the focus should then be on describing the extent of the lesions and the various organ involvements.

## Differential diagnosis and pitfalls for gynaecological venous malformations

GVMs, particularly when located in the myometrium, may be misdiagnosed as other vascular anomalies, including fast-flow uterine vascular anomalies (such as arteriovenous malformations, retained products of conception, uterine vascular tumours or gestational trophoblastic disease), as well as confounding venous anomalies like pelvic congestion syndrome or hormonally induced myometrium thickening. Careful imaging analysis, combined with clinical and biological correlation, is essential to ensure precise diagnosis and differentiation.

### Congenital arteriovenous malformations or acquired uterine arteriovenous fistulae

Arteriovenous malformations are considered congenital and typically present as complex pelvic vascular networks connecting uterine vessels to other pelvic vasculature, with multiple arteriovenous shunts [30]. In contrast, arteriovenous fistulae result from abnormal arteriovenous communications most often acquired following uterine trauma (e.g., dilation and curettage, operative hysteroscopy, caesarean section, etc.) and are usually confined to the myometrium and endometrium [30–34]. Serum hCG levels are consistently negative, aiding in their differentiation from other fast-flow uterine vascular anomalies [30, 31].

Unlike GVMs, arteriovenous malformations or fistulae are characterised by high-velocity, low-resistance arterial flow with aliasing on Doppler and early venous filling [30–32]. On MRI, they are composed of serpiginous T2 hypointense flow-voids and show intense arterial-phase enhancement, reflecting rapid arteriovenous shunting. A focal nidus with tortuous arteriovenous connections may also be seen [31]. These features are absent in GVMs, which lack flow-voids and exhibit gradual, delayed enhancement.

### Retained products of conception

Trophoblastic retention is a common condition following pregnancy or abortion, characterised by the persistence of chorionic villi, indicating trophoblastic or placental remnants [34]. It differs from other gestational trophoblastic diseases due to low or mildly elevated hCG levels, which normalise as the condition resolves. Patients often present with postpartum haemorrhage, pelvic pain, and rarely fever [34, 35].

Trophoblastic retention usually predominates or is confined to the uterine cavity and shows myometrial vessels with high-velocity, low-resistance flow on Doppler [30, 31, 34, 35]. On ultrasound, it appears as an echogenic intrauterine mass or thickening. MRI shows a heterogeneous intracavitary mass with variable but mostly early arterial enhancement [34, 35], with no infiltrative pattern. Indeed, adjacent vascular myometrial anomalies may only exhibit high-velocity flow of varying degrees, reflecting the myometrial vascularisation that supplies the trophoblastic remnants within the uterine cavity. Conversely, GVMs do not present as an intracavitary mass; instead, they show myometrial T2-hyperintense serpiginous veins and exhibit slow, progressive contrast uptake without early arterial enhancement.

### Gestational trophoblastic tumours (GTT)

Gestational trophoblastic tumours (GTT) are a rare group of disorders that encompass invasive mole, choriocarcinoma, or placental site trophoblastic tumours, and most often develop following a history of hydatidiform mole. GTT should be suspected in cases of unexplained postpartum bleeding lasting over 6 weeks with elevated hCG levels, after excluding a new pregnancy [34, 35].

On ultrasound and MRI, GTT may appear as intracavitary masses, but their hallmark is myometrial tumour infiltration with fast-flow vascular myometrial anomalies caused by tumoral arteriovenous shunts—10 to 15% of which may persist even after complete disease resolution (Fig. 10) [35]. In contrast, GVMs lack true flow-voids and exhibit slow, progressive contrast enhancement, without any solid component. Clinical and biological data, particularly elevated hCG levels, are useful to differentiate trophoblastic disease from GVMs.

### Gynaecological vascular haemangiomas

Haemangiomas are benign vascular tumours which are acquired proliferative lesions that fundamentally differ from venous malformations, which are congenital and non-proliferative. Haemangiomas typically arise postnatally, following a rapid proliferative phase and spontaneous involution [17]. They most often involve the cutaneous tissues of the head and neck (60%) [17, 29, 36]. Deeper or visceral locations may occur, such as intraglandular or intramuscular locations [5, 37], although the genital area is rarely affected [36].

**Fig. 11:**
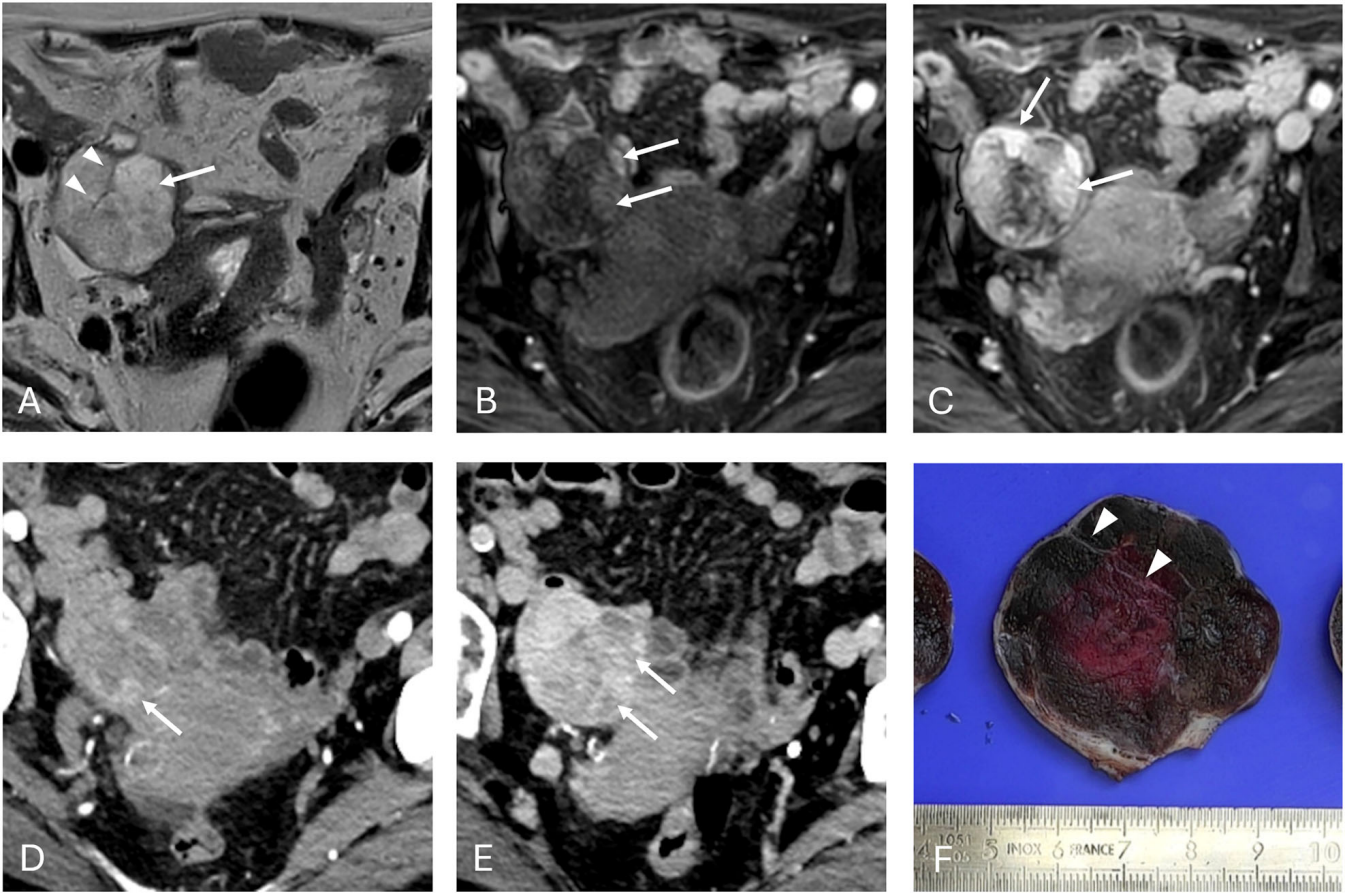
Incidental subserosal uterine vascular tumour in a 53-year-old patient initially admitted due to persistent haematuria. The patient had a history of renal angiomyolipoma treated by embolisation 3 years earlier and no gynaecological lesion on previous MRI or CT examinations. **A** Axial T2-W MR image shows a well-delineated, lobulated para-uterine lesion with high T2 signal intensity (arrows), and no sponge-like pattern. Note the presence of thin septa (arrowheads) and subtle internal signal heterogeneity. Diffusion-weighted imaging (DWI) was non-diagnostic due to artefacts (not shown). **B** Axial dynamic post-contrast T1-W MR fat-suppressed images at the arterial and **C** at the venous phase show early and sustained enhancement of the lesion, arguing against venous malformations (arrows). **D** Axial post-contrast CT images at the arterial and **E** at the venous phase show early and sustained enhancement of the lesion (arrows), and no intralesional calcifications. **F** Gross specimen photograph of the lesion shows reddish vascular tissue with visible septa (arrowheads), with a final diagnosis on microscopy of anastomosing haemangioma. No ectatic tubular vascular structures were identified

Gynaecological haemangiomas are extremely rare entities that show similar imaging features than other sites, appearing as well-defined, lobulated, homogeneous lesions (Fig. 11) [5, 17, 36, 37]. Only a few cases involving the female genital tract have been reported to our knowledge [36, 38]. On MRI, they present low-to-intermediate T1 and high T2 signal intensity, with intense, homogeneous arterial-phase enhancement and occasional flow-voids, but no arteriovenous shunting [17, 29, 36, 37]. In contrast, GVMs lack flow-voids and enhance with a slow and progressive pattern.

### Pelvic venous congestion syndrome (PCS)

Pelvic Congestion Syndrome (PCS) is first characterised and defined by chronic pelvic pain, exacerbated by standing, hormonal changes, or intercourse, predominantly affecting premenopausal women, with increased prevalence in multiparous individuals [39, 40]. It is estimated to affect 30% of the population, with symptoms often subsiding after menopause [40–42]. The pathophysiology is mainly linked to venous valve incompetence or structural abnormalities causing blood reflux (such as Nutcracker Syndrome or May-Thurner Syndrome), resulting in pelvic varices [39, 40, 42].

While PCS also involve slow-flow venous abnormalities, diagnosis compared to GVMs is most often unequivocal and is characterised by dilated para-uterine and gonadal veins. Multiple dilated veins are often bilateral, though asymmetrically enlarged on the left side, with clear accentuated reflux during Valsalva manoeuvres on sonography [39, 40, 42]. In cases of PCS with extensive venous ectasia, large, dilated veins can be located near the uterus or involve prominent subserosal veins. However, PCS typically lacks a mass-like appearance within the myometrium—the involved veins remaining subserosal (Fig. 6) [41]. Furthermore, veins are mostly in high signal but may also show flow-void depending on velocities. In contrast, GVMs exhibit a more well-defined, lobulated mass-like lesion with serpiginous vascular channels within the myometrium and no flow-void.

### Hormonally induced diffuse myometrial thickening

Changes in the uterine body may occur with age and hormonal status, especially in patients on hormonal contraceptives (Supplementary Fig. 3). Zonal anatomy is best assessed on T2-weighted MRI sequences [43]. In the absence of pathology, the outer myometrium typically demonstrates intermediate to high T2 signal intensity, due to the presence of thin myometrial veins arranged in a homogenous pattern. This signal may increase during the secretory phase, possibly due to oedema and higher water content within the myometrium [43, 44]. The junctional zone remains hypointense and shows minimal variation throughout the menstrual cycle, likely due to its lower water content [43, 45].

Elevated levels of oestrogen or progesterone can lead to a swollen appearance of the myometrium [46]. In this setting, the uterus maintains normal zonal anatomy, predominantly affecting the outer myometrium without significant thickening but with an increased signal intensity on T2-weighted MRI (resulting from myometrial hypertrophy, oedema, and sinusoidal dilatation), along with a thinned, sometimes indistinct inner myometrium (e.g., junctional zone) and a thin, atrophic endometrium [43, 46]. Higher-dose oral contraceptives are associated with more pronounced imaging features, but the appearance remains distinct from a GVM, as it involves the entire outer myometrium, preserves zonal anatomy including the junctional zone, and does not produce enlargement or a mass-like appearance [46].

## Patient management

Definitive diagnosis relies on histopathological analysis, obtained through targeted biopsy in cases of atypical presentation or to identify somatic mutations that may guide targeted therapies [2]. In certain cases, biopsy may be deferred when a robust clinico-radiological correlation allows for a confident diagnosis, particularly within expert vascular anomaly centres. This may include the presence of associated lesions, characteristic regional involvement suggesting a syndromic context, or radiological features deemed relatively pathognomonic. For example, a well-defined lesion of the female genital tract with preserved high T2 signal intensity on fat-suppressed sequences, absence of a solid tumour component and a progressive enhancement pattern is highly suggestive of the diagnosis [8]. In such scenarios—as is the practice in our institution—histological analysis is primarily pursued for therapeutic purposes, notably genotyping.

### Impact on reproductive health

Although patients with uterine venous malformation face a higher risk during pregnancy, it seems possible, with rigorous monitoring and a conservative approach, to achieve a successful pregnancy in patients who wish to conceive, with deliveries either by vaginal birth or caesarean section [13, 27]. However, the impact of this spectrum of pathology on fertility itself in these patients has, to our knowledge, not been reported in the literature.

### Treatments

Imaging plays a central role in the pre-treatment phase of GVMs, as it guides therapeutic decisions and helps avoid unnecessary or inappropriate treatments, particularly in women of reproductive age. Key radiological features to report include lesion location, extent (focal versus diffuse), size, enhancement pattern, potential venous drainage and its subtype, and pelvic extension suggestive of syndromic involvement.

**Fig. 12:**
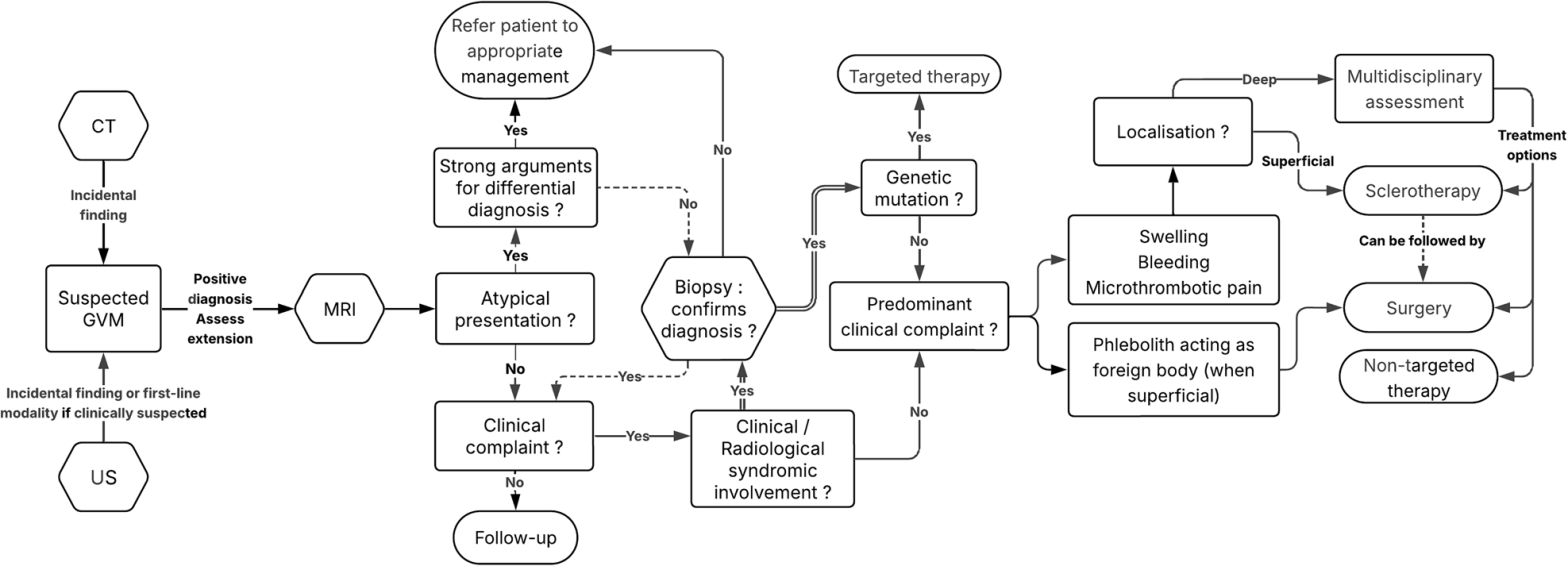
Diagnostic and treatment algorithm for gynaecological venous malformations. This flowchart outlines the different steps guiding the management of patients with suspected and/or confirmed gynaecological venous malformations. Management should be conducted in a specialised vascular anomalies centre, and patients should be referred following an MRI when the diagnosis is suspected. Biopsy may be deferred when there are strong clinical and radiological arguments supporting the diagnosis; however, it should be performed in cases of diagnostic uncertainty (dotted-arrow pathway) or for genotyping in syndromic forms (double-arrow pathway). A watchful waiting approach is recommended in the absence of clinical symptoms. In cases of deep involvement, due to potential fertility concerns, treatment decisions should be made through multidisciplinary discussion

Management of GVMs remains poorly defined due to their rarity [2, 47]. It is mainly guided by the lesion location, symptom severity, functional impairment or aesthetic impact, and pregnancy plans. Thus, referral to expert centres with tailored strategies is strongly recommended [14, 28]. We propose a diagnostic and treatment algorithm based on protocols used in our specialised centre (Fig. 12).

In asymptomatic or mildly symptomatic cases, a watchful waiting approach is appropriate [12, 48]. Avoidance of oestrogen-based hormonal contraception is advised, as it may promote the expansion of dysplastic venous lakes [25]. When treatment is necessary, options include surgical, interventional radiology, or pharmacological approaches tailored to lesion location and genomic subtype.

Percutaneous sclerotherapy is the gold standard for cutaneous venous malformations [29] and is often used with favourable outcomes for vulvo-vaginal or cervical GVMs [9, 49, 50]. In this context, understanding the venous connections between the GVM and the normal systemic venous network is critical for evaluating the feasibility and potential risks of endovascular treatment [16]. Indeed, the type of venous drainage influences both the risk and likelihood of success with sclerotherapy: the presence of a draining vein increases the risk of treatment-related toxicity [16, 29] or deep vein thrombosis [17].

Local surgical excision may be performed for smaller-sized vulvo-vaginal or cervical lesions, preferably following sclerotherapy to reduce lesion size and intraoperative bleeding risk [3]. Other reported conservative treatments include CO₂ laser excision, cryotherapy, electrocauterization, internal artery ligation, uterine artery embolisation, and laser ablation [2, 10, 12, 28, 47].

In cases of deeper GVMs, particularly in the uterine corpus, or in syndromic lesions with extensive involvement, limited endovascular access may warrant the use of anti-angiogenic pharmacological therapies. They can be targeted (e.g., alpelisib or miransertib) if genotyping is positive, or non-targeted (such as sirolimus) otherwise [51]. Sirolimus has been widely used for several years in certain cases of cutaneous venous malformations and has proven effective in reducing lesion size [48, 52–54]. Although not specifically studied for GVMs, similar pathophysiology suggests potential applicability for these treatments.

In severe or refractory cases involving deep lesions, hysterectomy may be considered based on patient age and pregnancy plans [2, 3, 10, 12, 28, 47].

### Follow-up

Monitoring of gynaecological venous malformations is essential, as spontaneous growth or recurrence after treatment is common, especially during puberty and pregnancy [12]. In the absence of clinical symptoms and prior treatment, we recommend annual follow-up with ultrasound and/or MRI, depending on lesion location.

Post-treatment follow-up requires precise documentation of lesion volume to accurately assess therapeutic response, as the largest axis commonly used in tumour monitoring is not relevant for venous malformations due to their non-spherical shape in both isolated and syndromic involvement [51, 55].

A delay of up to several months is necessary to evaluate therapeutic response after sclerotherapy, allowing time for the transient inflammatory response to resolve [17]. However, immediate post-treatment MR imaging can show high signal intensity in the treated areas on T2-weighted and STIR images, up to 3 months after treatment, and intense peripheral hyperenhancement secondary to reactive hyperaemia [17].

Satisfactory long-term post-treatment MRI findings show decreased T2 and T2 Fat Sat signal intensity and significant lesion size reduction (Fig. 9), along with improvement or resolution of the patient’s symptoms on clinical assessment [12, 17].

However, no specific data exists for GVM follow-up after treatment. In our centre, efficacy after sclerotherapy is assessed via a follow-up visit combined with an ultrasound and/or MRI 3 months after treatment.

## Conclusion

Radiological imaging is central in the diagnosis, management, and follow-up of GVMs, while also enhancing the understanding of this rare condition. Key challenges include accurate diagnosis, exclusion of mimicking conditions, and identification of syndromic involvement to ensure referral to specialised centres. Further research is needed to clarify pathophysiology, standardise imaging protocols, and define diagnostic hallmarks. Critical literature review is essential, as outdated terminology persists, impeding classification harmonisation, progress in research and patient care.

## Supplementary information

ELECTRONIC SUPPLEMENTARY MATERIAL

## Data Availability

All patients’ clinical and radiological data were from Lyon Sud University Hospital and Lyon Femme-Mère-Enfant University Hospital.

## Abbreviations

AVMs: Arteriovenous malformations
CLOVES Syndrome: Congenital, lipomatous overgrowth, vascular malformations, epidermal nevi, scoliosis/skeletal/spinal anomalies syndrome
GTT: Gestational trophoblastic tumours
GVMs: Gynaecological venous malformations
hCG: Human chorionic gonadotrophin
PCS: Pelvic congestion syndrome
PROS: PIK3CA-related overgrowth syndrome
VMCM: Cutaneous and mucosal venous malformations
