# Supplementary material for: Slow-flow, high-impact: radiologic features in venous malformations of the female genital tract
Source: Insights Imaging. 2026 Jun 30;17:179. doi: 10.1186/s13244-026-02343-z (PMC13319296; doi:10.1186/s13244-026-02343-z)

Slow-Flow, High-Impact: Radiologic Features in Venous Malformations of the Female Genital Tract

ELECTRONIC SUPPLEMENTARY MATERIAL

Insights Imaging (2026) Guihard M, Fraissenon A, Collardeau-Frachon S, et al.

Supplemental – Fig. 1 – Gynaecological venous malformation (*GVM) of the right labia majora in a 6-year-old girl presenting with local swelling and pain.*

(A) Suprapubic ultrasound image shows a well-defined, heterogeneous subcutaneous lesion (*arrows*), with hypoechoic tubular structures corresponding to dilated veins (*dotted arrows*).

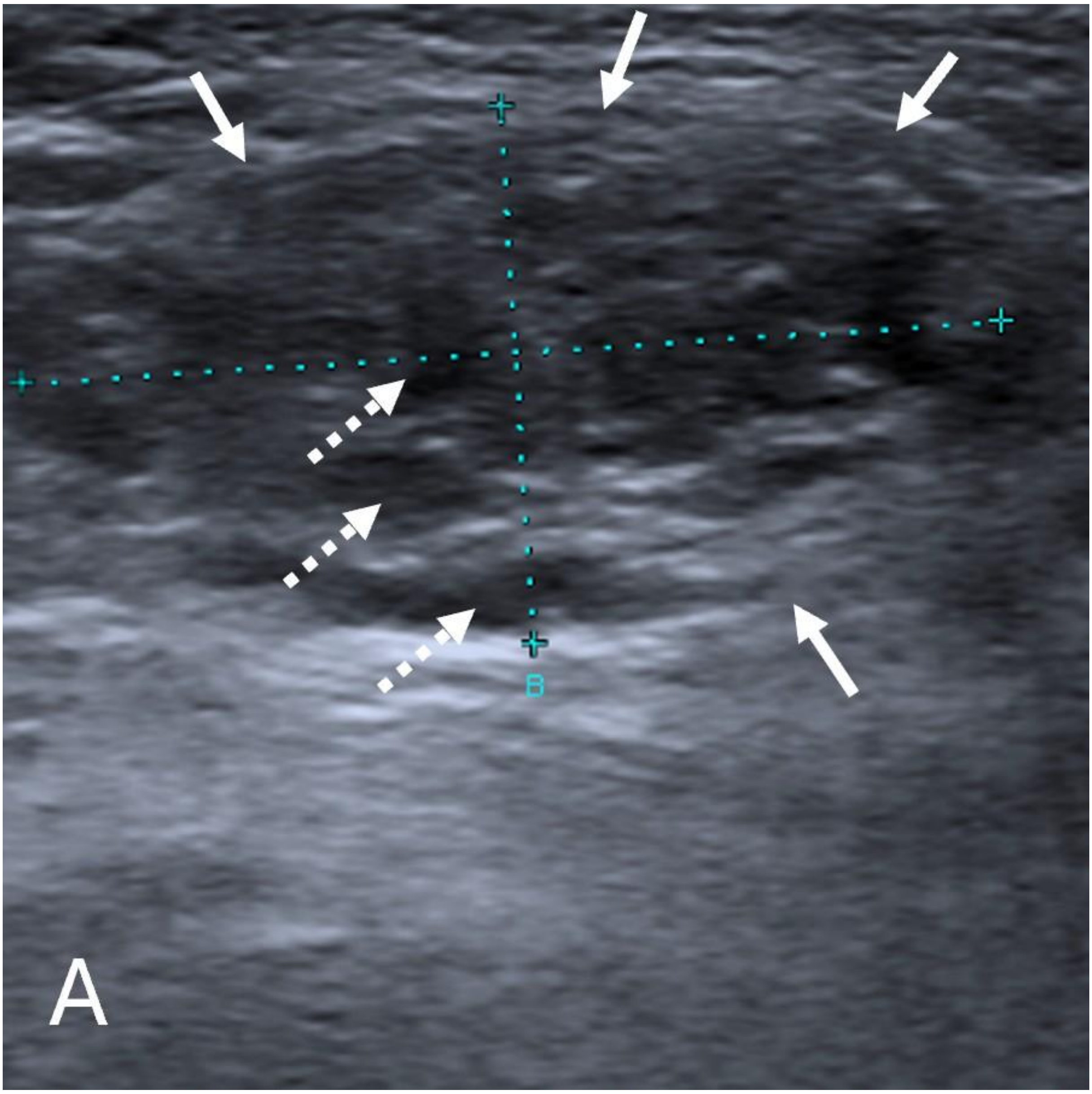

(B) Axial and **(C)** sagittal T2-W fat-suppressed image shows a well-defined lesion of the right labia majora, with hyperintense T2W serpiginous structures and absence of flow voids (*arrows*).

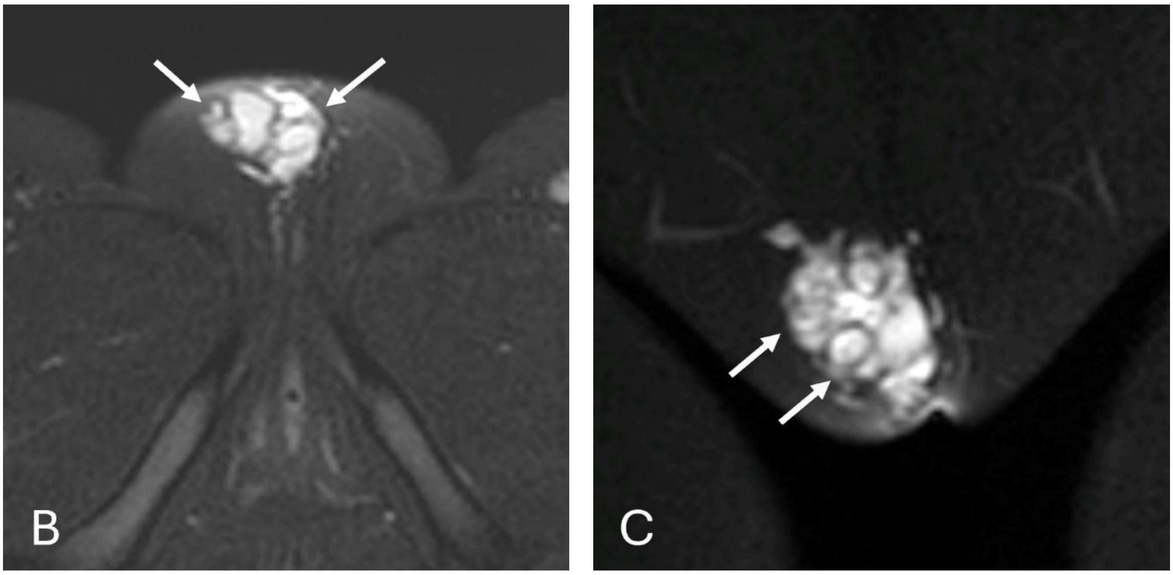

(D) Coronal image of transperineal venography images before and **(E)** after percutaneous sclerotherapy using sclerosing agent injection show a venous malformation with ectatic dysplastic intralesional veins (*arrows*) and no venous drainage to the systemic network. As a transperineal procedure, a needle was directly inserted within the dysplastic vessels of the lesion (*black arrowhead*). Post-injection image shows a satisfactory result with no residual blood flow (*dotted arrow*). Percutaneous sclerotherapy was performed one month after the acute episod.

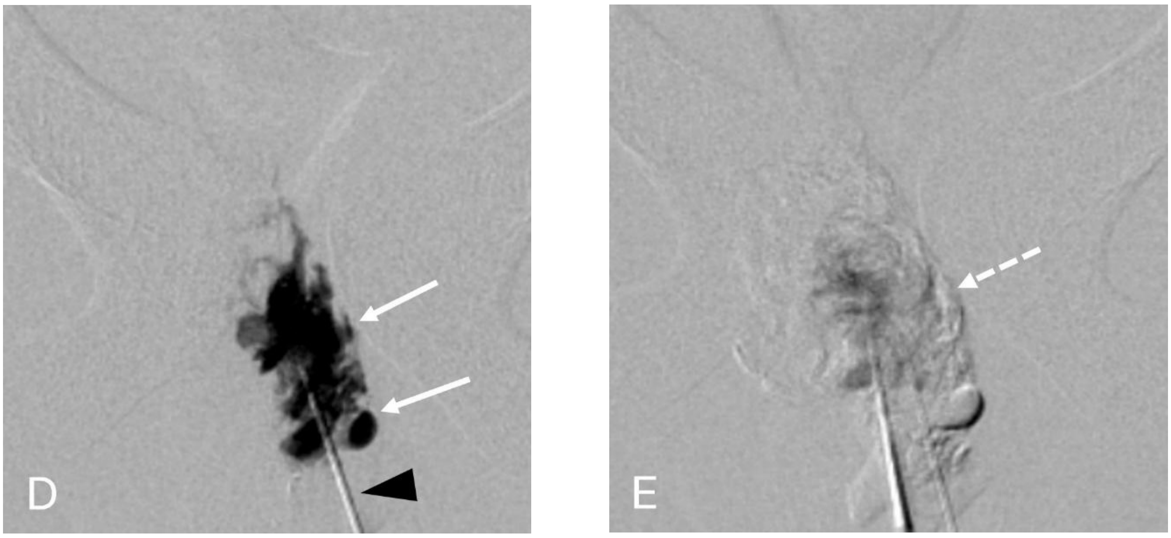

(F) Axial T2-W fat-suppressed MR image obtained at five-month posttreatment follow-up shows a marked decrease in lesion size and signal intensity (*arrows*).

The residual lesion was later excised surgically for cosmetic reasons.

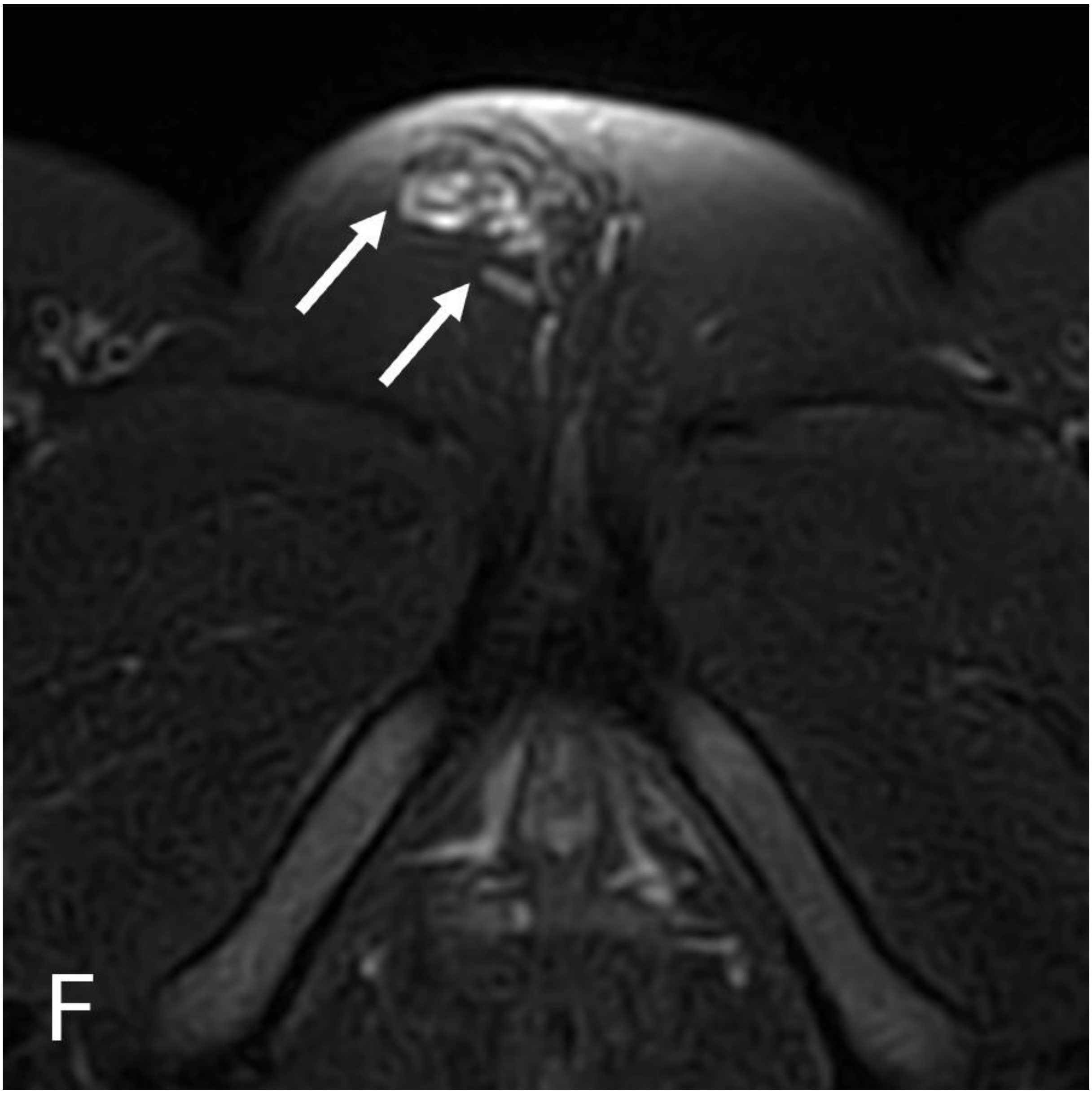

Supplemental – Fig. 2 – *Extensive venous malformations involving the female genital tract in a 23-year-old patient with PROS syndrome and a PIK3CA mutation, treated with alpelisib.*

(A, B) Axial T2-W fat-suppressed MR images show diffuse T2-hyperintense thickening of the uterine corpus and cervix (*arrows*), partially obscuring the zonal anatomy with a blurred junctional zone (*arrowheads*). Note the additional extensive contiguous infiltration of the venous malformation of the right parametrium, pelvic wall, vulva, and perineum (*dotted arrows*).

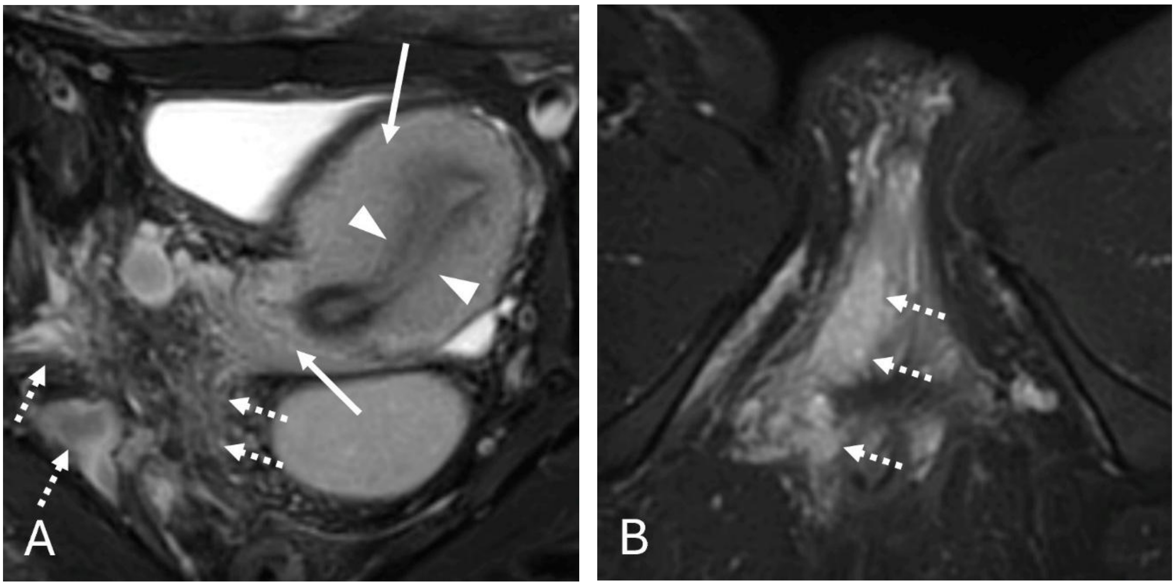

(C) Axial T2-W fat-suppressed MR image shows an involvement of the right lower limb, as often observed in syndromic PROS cases, with serpiginous dilated T2-hyperintense venous structures within the subcutaneous tissues (*dotted arrows*), an absence of flow voids, and an enlargement of the affected thigh.

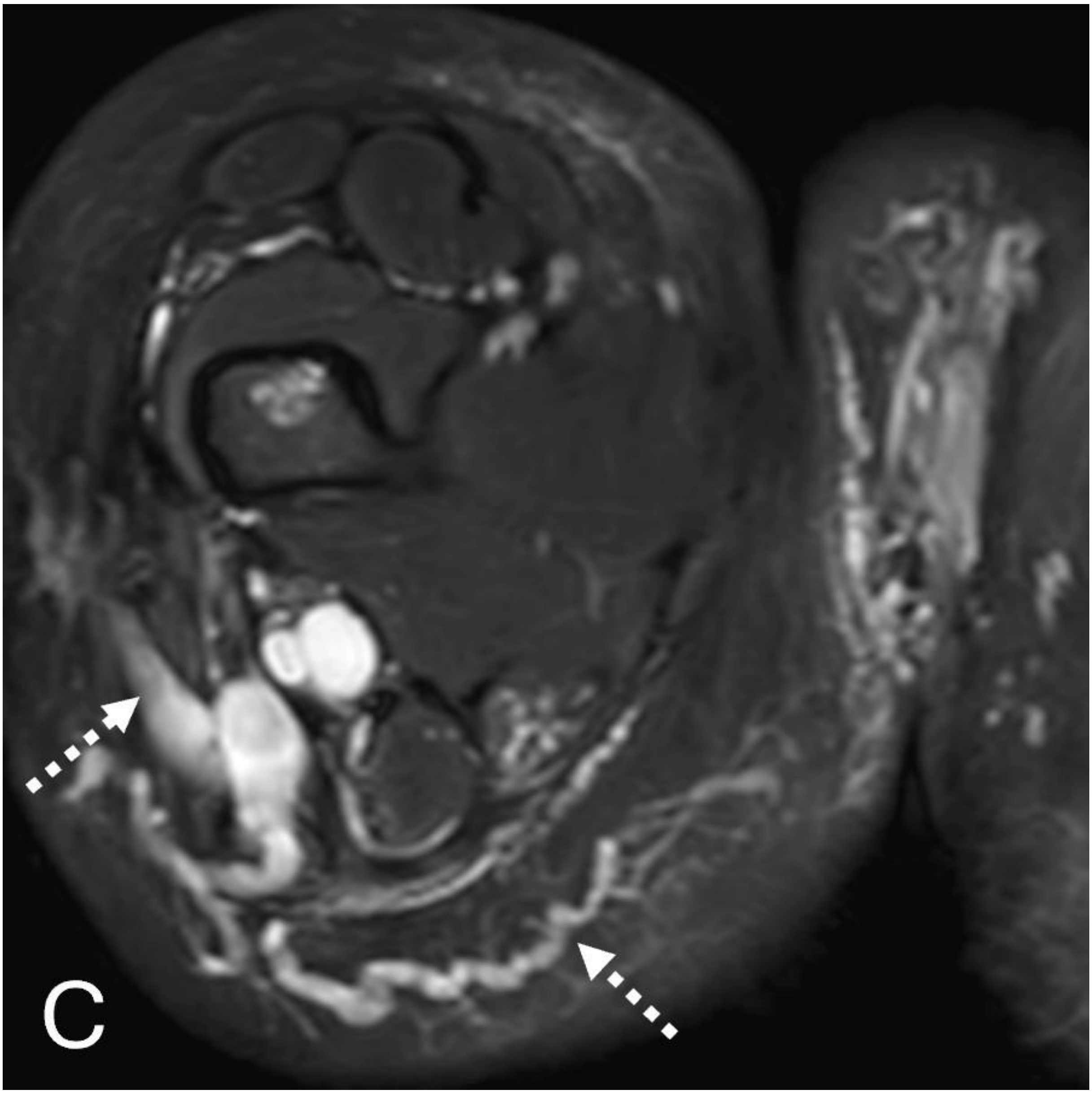

(D) Axial non-contrast CT image shows an isodense infiltrative venous malformation of the vulva (*arrows*), with scattered calcifications (*arrowheads*), consistent with phleboliths within the venous malformation.

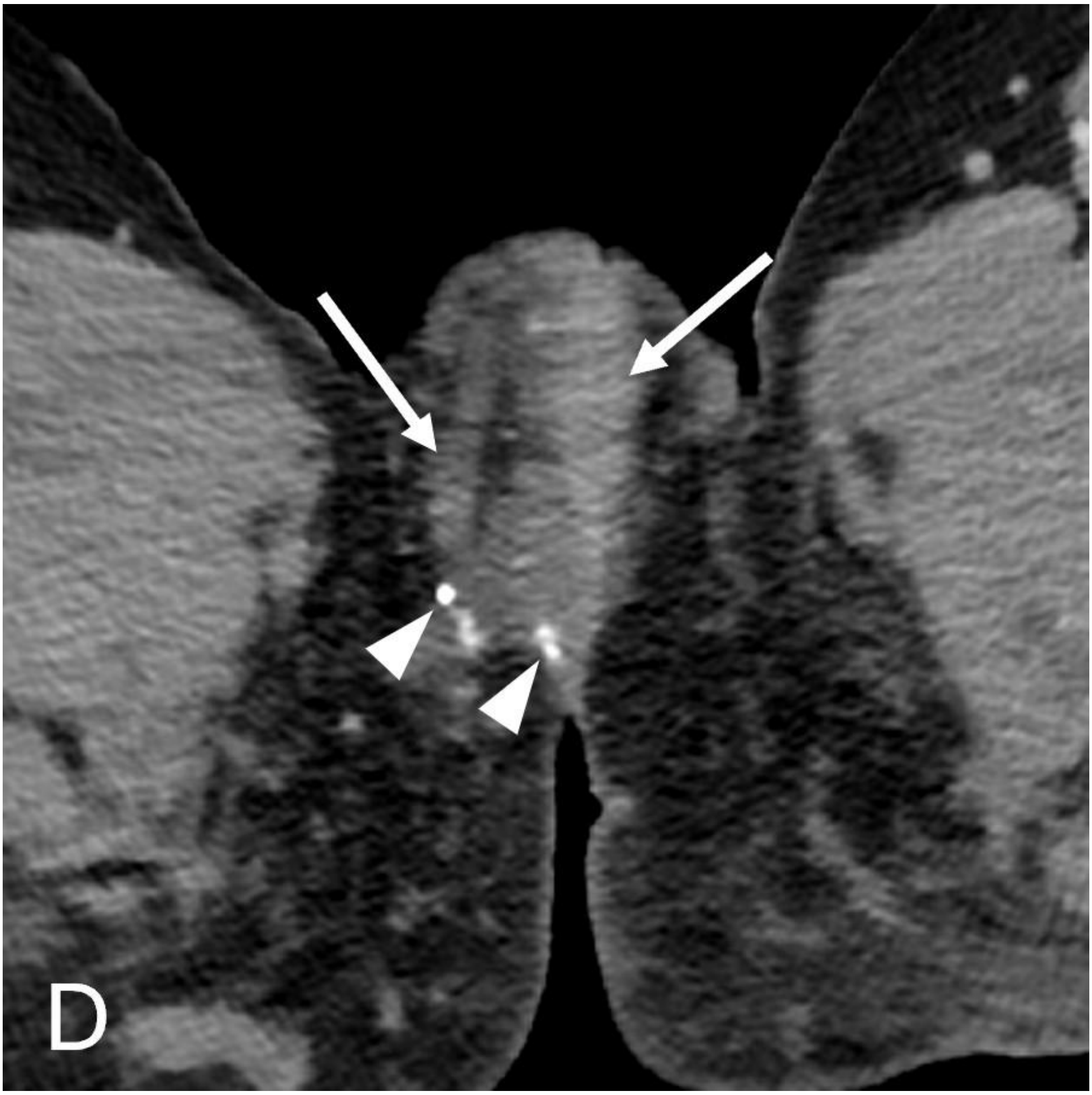

Supplemental – Fig 3 – *Myometrial signal changes on oral contraception in a 33-year-old patient, presenting with severe dysmenorrhea and digestive complaints while on continuous oral contraception (drospirenone) for suspected endometriosis.*

(A) Axial T2-W MR image shows diffuse myometrial T2 hyperintensity (*arrows*) due to outer myometrial veins, without flow voids, mass effect or objective thickening, with clear depiction of the junctional zone (*arrowheads*).

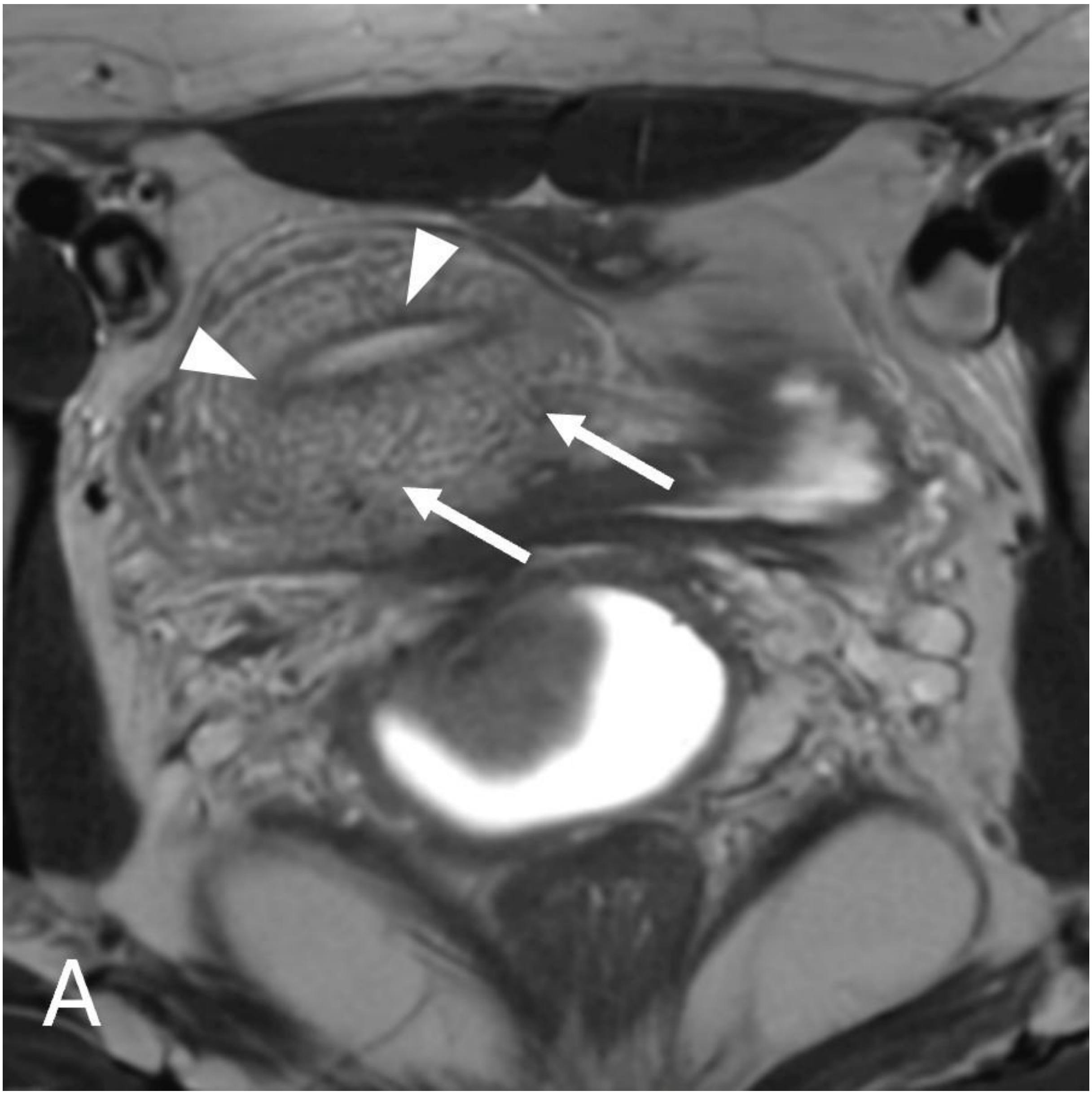

(B, C) Sagittal T2-W MR images demonstrate a normal-sized uterus with preservation of the junctional zone (*arrowhead*) and continuity between the outer myometrium and the outer cervical stroma, physiological at this age, without any sharply delineated signal or morphological changes (*arrows*). Of note, outer myometrial veins are of uniform caliber, with a homogeneous pattern. Note also the typical uterine contraction appearing as an ill-defined, low T2 signal area (*dotted arrow*) perpendicular to the junctional zone, consistent with functional outer myometrium.

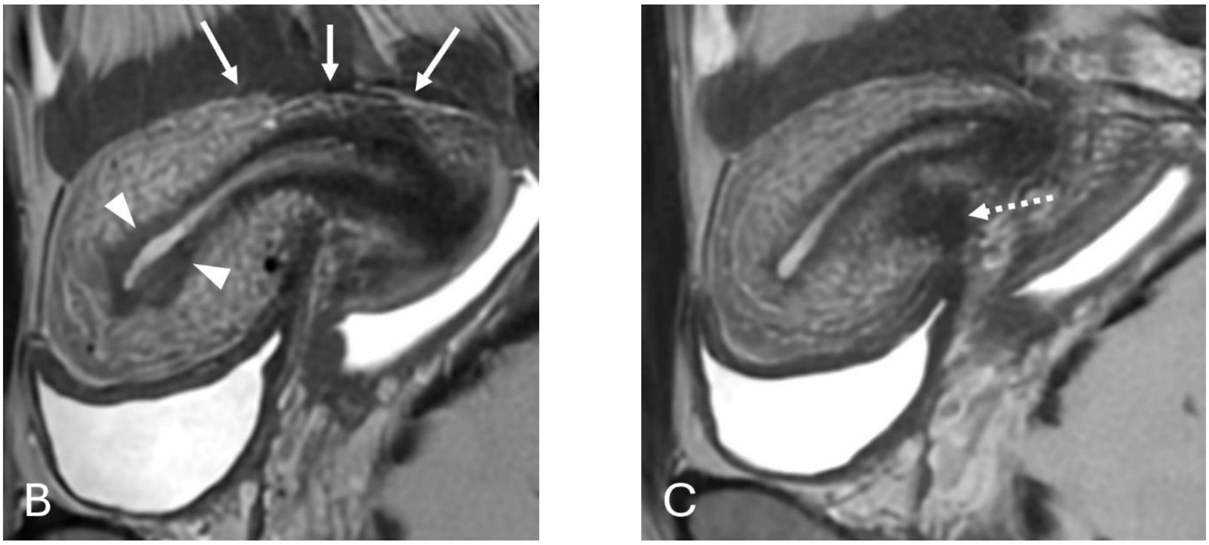

Supplement: Supplementary file 1 — ELECTRONIC SUPPLEMENTARY MATERIAL [file 13244_2026_2343_MOESM1_ESM.pdf]
